# Therapeutic utility of natural estrogen receptor beta agonists on ovarian cancer

**DOI:** 10.18632/oncotarget.18442

**Published:** 2017-06-12

**Authors:** Jinyou Liu, Suryavathi Viswanadhapalli, Lauren Garcia, Mei Zhou, Binoj C. Nair, Edward Kost, Rajeshwar Rao Tekmal, Rong Li, Manjeet K. Rao, Tyler Curiel, Ratna K. Vadlamudi, Gangadhara R. Sareddy

**Affiliations:** ^1^ Department of Obstetrics and Gynecology, Cancer Therapy and Research Center, University of Texas Health Science Center, San Antonio, Texas, USA; ^2^ Department of Molecular Medicine, University of Texas Health Science Center, San Antonio, Texas, USA; ^3^ Department of Cell Systems and Anatomy, University of Texas Health Science Center, San Antonio, Texas, USA; ^4^ Department of Medicine, University of Texas Health Science Center, San Antonio, Texas, USA; ^5^ Department of Oncology, Xiangya School of Medicine, Central South University, Hunan, P.R. China; ^6^ Department of Gastroenterology, Second Xiangya Hospital and Xiangya School of Medicine, Central South University, Hunan, P.R. China

**Keywords:** ovarian cancer, natural ERβ agonists, NF-κB

## Abstract

Ovarian cancer is the deadliest of all gynecologic cancers. Despite success with initial chemotherapy, the majority of patients relapse with an incurable disease. Development of chemotherapy resistance is a major factor for poor long-term survival in ovarian cancer. The biological effects of estrogens are mediated by estrogen receptor alpha (ERα) and estrogen receptor beta (ERβ). Emerging evidence suggests that ovarian cancer cells express ERβ that functions as a tumor suppressor; however, the clinical utility of ERβ agonists in ovarian cancer remains elusive. We tested the utility of two natural ERβ agonists liquiritigenin (Liq), which is isolated from *Glycyrrhiza uralensis* and S-equol, which is isolated from soy isoflavone daidzein, for treating ovarian cancer. Both natural ERβ ligands had significant growth inhibition in cell viability and survival assays, reduced migration and invasion, and promoted apoptosis. Further, ERβ agonists showed tumor suppressive functions in therapy-resistant ovarian cancer model cells and sensitized ovarian cancer cells to cisplatin and paclitaxel treatment. Global RNA-Seq analysis revealed that ERβ agonists modulate several tumor suppressive pathways, including downregulation of the NF-κB pathway. Immunoprecipitation assays revealed that ERβ interacts with p65 subunit of NF-κB and ERβ overexpression reduced the expression of NF-κB target genes. In xenograft assays, ERβ agonists reduced tumor growth and promoted apoptosis. Collectively, our findings demonstrated that natural ERβ agonists have the potential to significantly inhibit ovarian cancer cell growth by anti-inflammatory and pro-apoptotic actions, and natural ERβ agonists represent novel therapeutic agents for the management of ovarian cancer.

## INTRODUCTION

Ovarian cancer (OCa) is the leading cause of death from gynecologic malignancies [1, 2]. Due to the lack of early stage markers, advanced stage diagnosis of OCa is common and majority of patients will relapse with incurable disease despite therapy [3]. The lethality of OCa primarily stems from our inability to detect the disease at an early, organ-confined stage and also due to the lack of effective therapies for advanced disease [4, 5]. Further, the molecular basis of this disease is not completely understood. This represents a significant problem in the management of patients with OCa and a critical need for the development of novel therapies treating OCa.

Estrogens are the main female sex steroid hormones that play a critical role in the proliferation and differentiation of ovary [6]. The biological effects of estrogens are mediated by estrogen receptor alpha (ERα) and estrogen receptor beta (ERβ) [7, 8]. ERβ functions as a tissue-specific tumor suppressor with anti-proliferative actions [8, 9]. ERβ is a transcription factor that is implicated in the modulation of genes involved in multiple tumor suppression–related pathways. ERβ tumor suppression functions may be mediated by both ERβ:ERβ homodimer (that activate tumor suppressor functions) and ERβ:ERα heterodimer (that blocks ERα oncogenic signaling) depending on the status of the cellular expression of ERs [10–12]. However, the molecular mechanism(s) through which ERβ mediates growth inhibition of OCa cells remains elusive.

Even though ERα and ERβ are structurally similar, their ligand-binding domains differ enough to be selective for different ligands [8, 13]. Recent studies identified liquiritigenin (Liq) [14] and S-equol as ERβ-specific agonists [15, 16]. Liq is isolated from the plant *Glycyrrhiza uralensis* and is a potent ERβ agonist; Liq exhibits a 20-fold higher affinity for ERβ than for ERα [14]. S-equol is a compound that was isolated from the soy isoflavone daidzein via biotransformation [17]. S-equol has preferential binding to ERβ (Ki of 0.73 nM for ERβ compared to Ki of 6.41 nM for ERα) and functions as an ERβ agonist [17, 18]. Currently, Liq and S-equol are being tested to treat vasomotor symptoms (hot flashes) associated with menopause [12, 18]. In Phase II clinical trials, Liq and S-equol were found to be safe and well tolerated and taken with high compliance.

In this study, we tested the significance and therapeutic potential of ERβ signaling in OCa progression using natural ERβ agonists as novel therapeutic agents. Using both *in vitro* and *in vivo* models, we demonstrated that natural ERβ agonists have tumor suppressive functions on OCa cells. Mechanistic studies showed that ERβ agonists modulate several tumor inhibitory and inflammatory pathways, including attenuation of the NF-κB pathway. Further, treatment with ERβ agonists reduced *in vivo* tumor growth and promoted apoptosis in a xenograft model.

## RESULTS

### Natural ERβ agonists reduced cell viability and survival and promoted apoptosis of OCa cells

**Figure 1:**
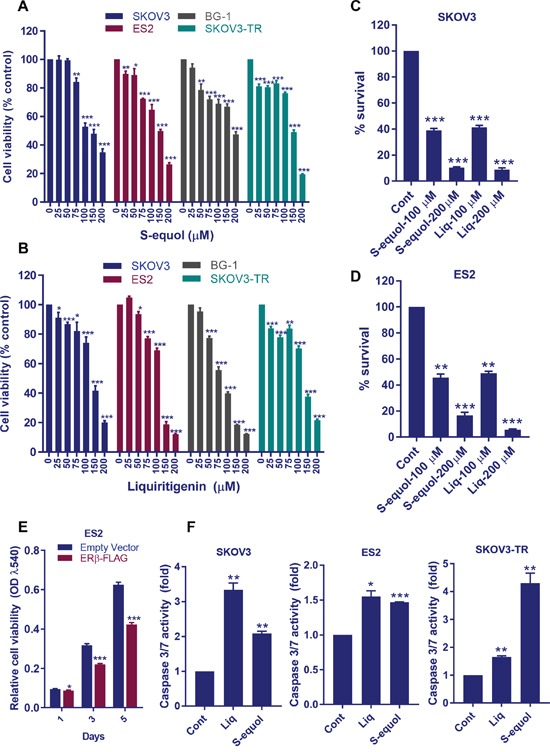
ERβ agonists reduce OCa cell viability, survival and promote apoptosis *in vitro* **(A-B)** ES2, BG-1, SKOV3, and SKOV3-TR cells were treated with vehicle or S-equol or Liq for 72 h, and the cell viability was measured by using a MTT assay. **(C-D)** ES2 and SKOV3 cells were treated with vehicle, S-equol, or Liq for 72 h and then cultured in growth medium for additional 7 days. The number of colonies for each group was counted. **(E)** ES2 cells were transduced with either empty or ERβ-FLAG expression vector and cell proliferation rates were measured using MTT assay. **(F)** ES2, SKOV3, and SKOV3-TR cells were treated with vehicle, S-equol, or Liq for 48 h, and the Caspase-3/7 activity was measured as described in Methods. Data are represented as mean ± SE. * p<0.05; ** p<0.01; *** p<0.001.

We examined whether activation of ERβ pathway by its natural agonists contribute to the reduction of cell viability and survival of OCa cells. SKOV3 and BG1 cells treated with S-equol or Liq exhibited a significant dose-dependent reduction in viability (Figure 1A, 1B). Further, treatment with S-equol and Liq also exhibited an inhibitory effect on the viability of therapy-resistant ES2 (cisplatin resistant) and SKOV3-TR (taxol resistant) cells (Figure 1A, 1B). These natural ERβ agonists significantly reduced the colony formation ability of ES2 and SKOV3 cells (Figure 1C, 1D). To further confirm the effect of ERβ on cell proliferation, ES2 cells were transduced with ERβ expression vector. Overexpression of ERβ significantly reduced the proliferation of ES2 cells when compared to control cells (Figure 1E). We next examined whether ERβ agonists can induce apoptosis as measured by caspase 3/7 activity. As shown in Figure 1F, both S-equol and Liq significantly increased the caspase 3/7 activity in ES2, SKOV3 and SKOV3-TR cells. Collectively, these results suggest that natural ERβ agonists have the potential to reduce cell viability and survival and to promote apoptosis of OCa cells.

### ERβ agonist sensitized OCa cells to chemotherapeutic drugs

**Figure 2:**
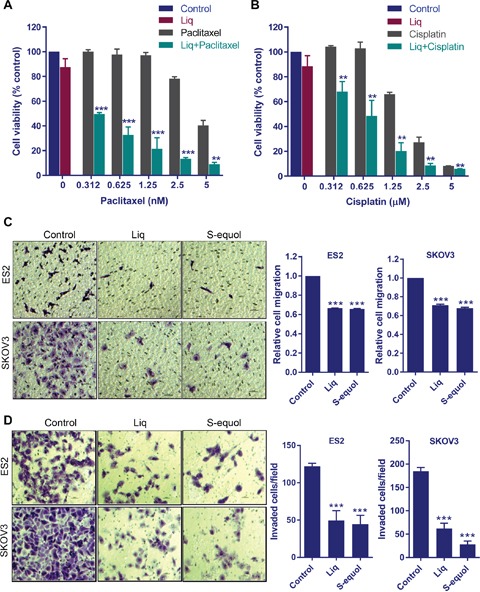
ERβ agonists sensitize OCa cells to paclitaxel and cisplatin treatment and reduce migration and invasion of OCa cells **(A-B)** ES2 cells were pretreated with Liq (25 μM) for 48 h followed by treatment with varying doses of cytotoxic drugs paclitaxel or cisplatin for an additional 5 days. Cell viability was determined using MTT assay. **(C)** ES2 and SKOV3 cells were treated with vehicle, Liq or S-equol for 24 h and then used in transwell migration assays. Optical density was measured 16 h after migration. Photomicrographs of migrated cells in various treatments are shown. **(D)** Cell invasion potential of ES2 and SKOV3 cells treated with ERβ agonists was analyzed by using Matrigel invasion chamber assays. Photomicrographs of invaded cells in various treatments are shown. Data are represented as mean ± SE. ** p<0.01; *** p<0.001.

The standard chemotherapy treatment for OCa patients include the platinum based therapy or combination of paclitaxel with a platinum based compounds. Emerging studies suggested that using a multi-targeted approach has an advantage over using a single agent for OCa therapy, and sensitizing agents that improve the efficacy of chemotherapy are beneficial [19, 20]. Since, ERβ agonists reduce the cell viability of therapy sensitive and resistant cells, we next examined whether ERβ agonist sensitizes the OCa cells to standard of care chemotherapy. ES2 and SKOV3 cells were pre-treated with Liq followed by treatment with chemotherapeutic drugs cisplatin and paclitaxel. ERβ agonist Liq significantly sensitized OCa cells to paclitaxel and cisplatin treatment (Figure 2A, 2B).

### Natural ERβ agonists reduced migration and invasion of OCa cells

Recent studies using overexpression of ERβ demonstrated that reintroduction of ERβ into epithelial ovarian cancer cells leads to reduced cell proliferation, improved survival of mice and decreased metastases [21]. To examine the effect of natural ERβ agonists in reducing the migration and invasion of OCa cells, we performed *in vitro* migration and invasion assays. Treatment with either S-equol or Liq resulted in significantly less migration for both the SKOV3 and ES2 cells than for vehicle-treated cells (Figure 2C). Further, treatment with either S-equol or Liq also significantly reduced the ability of ES2 and SKOV3 cells to invade the matrigel (Figure 2D). Collectively, these results suggested that natural ERβ agonists have the potential to reduce migration and invasion of OCa cells.

### Natural ERβ agonists modulated expression of genes that contribute to tumor progression

**Figure 3:**
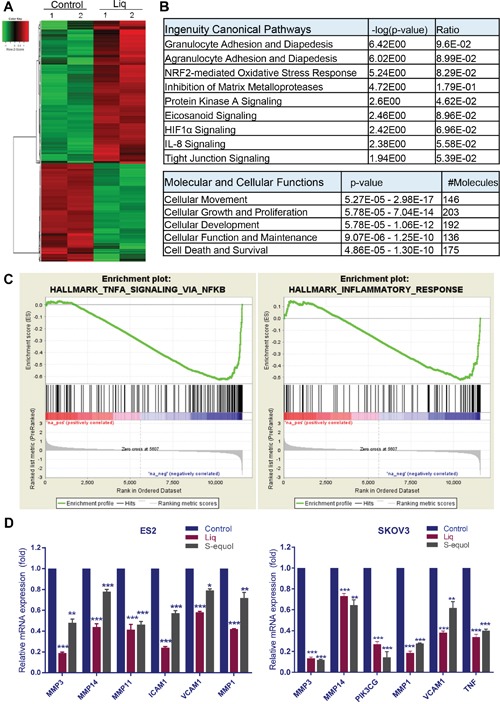
Analysis of global transcriptional changes modulated by ERβ agonists in OCa cells Total RNA was isolated from the ES2 cells that were treated with either vehicle or Liq (100 μM) for 24 h and subjected to RNA sequencing. **(A)** Heat map of differentially expressed genes between vehicle and Liq is shown. **(B)** Differentially expressed genes were subjected to pathway analysis using IPA software, and the selected top canonical pathways are shown. Analysis of molecular and cellular functions of differentially expressed genes are shown. **(C)** Gene set enrichment analysis (GSEA) testing correlation of Liq-regulated genes with signatures of NF-κB signaling gene set and inflammatory response gene set. **(D)** ES2 and SKOV3 cells were treated with either vehicle or Liq or S-equol for 24 h, and the selective genes representing each pathway were validated by using RT-qPCR. Data are represented as mean ± SE. * p<0.05; ** p<0.01; *** p<0.001.

To understand the mechanisms by which natural ERβ agonists promote tumor suppression, we performed RNA sequencing to examine the gene expression changes modulated by Liq. ES2 cells were subjected to treatment with either vehicle or Liq for 24 h. RNA was isolated and used for a global transcriptome analysis. Overall, 525 genes (1.5 fold change over control with *P* < 0.05) were differentially expressed in Liq–treated cells; 214 genes were downregulated and 311 genes were upregulated. A representative heat map of differentially expressed genes is shown in Figure 3A. The complete list is available in the GEO database under accession number GSE93807. The biological significance of the differentially expressed genes was determined using Ingenuity Pathway Analysis (IPA). IPA analysis revealed that Liq treatment significantly modulated the genes involved in inflammation, NRF2-mediated oxidative stress response, and MMP signaling (Figure 3B). Analysis of the molecular and cellular functions of these differentially expressed genes revealed that they are involved in cellular movement, cellular growth, proliferation, cell death and survival (Figure 3B). Further gene set enrichment analysis (GSEA) confirmed that the Liq-downregulated genes correlated well with signatures of inflammation, and NF-κB pathways (Figure 3C). Using RT-qPCR analyses, we validated the expression of selected genes, including pro-inflammatory cytokines, MMPs, and adhesion molecules in ES2 and SKOV3 cells (Figure 3D).

### Natural ERβ agonists attenuated activation of NF-κB pathway in OCa cells

**Figure 4:**
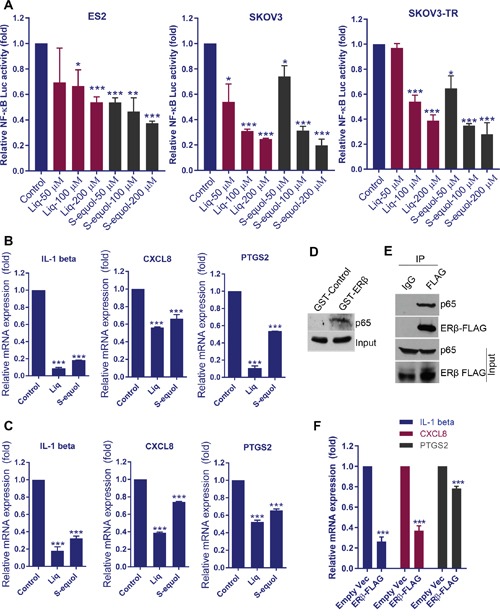
ERβ agonists attenuate NF-κB pathway activation **(A)** ES2, SKOV3, and SKOV3-TR cells were transfected with NF-κB-luc reporter plasmid and grown for 24 h. Then, cells were treated with ERβ agonists and reporter activity was measured after 24 h. ES2 **(B)** and SKOV3 **(C)** cells were treated with Liq or S-equol (100 μM) for 24 h, and the expression status of NF-κB target genes was analyzed by using RT-qPCR. **(D)** ES2 cell lysates were used in pull-down assays using GST or ERβ-GST, and interaction of ERβ with p65 was analyzed by Western blotting. **(E)** ES2-ERβ-FLAG cell lysates were subjected to immunoprecipitation with IgG or FLAG antibodies and the interaction of ERβ with p65 was confirmed by Western blotting. **(F)** SKOV3 cells were transduced with empty vector or ERβ-FLAG expression vector and the expression of NF-κB target genes was analyzed by using RT-qPCR. Data are represented as mean ± SE. * p<0.05; ** p<0.01; *** p<0.001.

NF-κB signaling plays a critical role in many steps of cancer initiation and progression and is a critical mediator of inflammatory responses [22]. Since RNA-seq data revealed that NF-κB and immune pathways are the top pathways modulated upon Liq treatment, we hypothesized that ERβ cross talk and inhibit activation of NF-κB via non-classical mechanisms. We treated cells with either S-equol or Liq and found that both agonists significantly reduced the NF-κB-Luc reporter activity in ES2, SKOV3 and SKOV3-TR cells in a dose-dependent manner (Figure 4A). Further, we confirmed the downregulation of the NF-κB target genes IL-1 beta, CXCL8 and PTGS2 by S-equol and Liq in ES2 and SKOV3 cells (Figure 4B, 4C). To demonstrate ERβ crosstalk with NF-κB pathway, we performed immunoprecipitation assays. GST pull-down assay using ERβ-GST revealed that ERβ interacts with p65 subunit of NF-κB (Figure 4D). Further, we also confirmed the interaction of ERβ with p65 by performing immunoprecipitation assays using ERβ overexpressed cells (Figure 4E). Importantly, overexpression of ERβ significantly reduced the expression of NF-κB target genes in SKOV3 cells (Figure 4F). These results suggest that ERβ interacts with NF-κB complex and attenuates the activation of NF-κB pathway.

### Natural ERβ agonist Liq reduced OCa tumor growth *in vivo*

**Figure 5:**
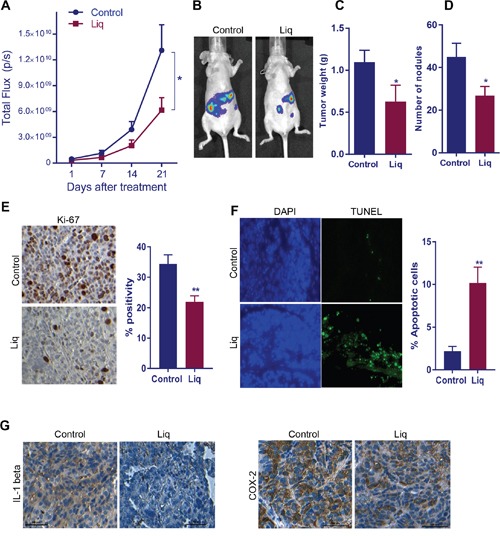
Effect of ERβ agonist Liq on OCa progression *in vivo* **(A)** Athymic nude mice were implanted with SKOV3ip1-Luc cells intra-peritoneally and treated with vehicle or Liq for 3 weeks. Luciferase intensity detected by the Xenogen *in vivo* imaging system was used to measure tumor growth. **(B)** Representative images of tumor-bearing mice from control and treatment groups are shown. The tumor weight **(C)** and number of nodules **(D)** from control and treatment group are shown. Ki67 expression **(E)** as a marker of proliferation and TUNEL staining **(F)** as a marker of apoptosis were analyzed by performing immunohistochemistry (IHC) on tumor sections. For quantitation, Ki-67-positive and apoptotic cells from five different fields were counted and plotted as histogram. Data are represented as mean ± SE. * p<0.05. **p<0.01. **(G)** The expression of NF-κB target genes IL-1 beta and Cox-2 was determined on vehicle or Liq treated tumor sections by performing IHC.

To complement *in vitro* studies, we tested the efficacy of the natural ERβ agonist Liq *in vivo* using OCa xenograft tumor model. SKOV3ip1-Luc cells were injected into the peritoneum of nude mice (n=5), and tumor growth was measured by using Xenogen *in vivo* imaging system. Liq treatment (20mg/Kg/day/Oral) significantly reduced tumor volume and tumor weight (Figure 5A-5C). Further, mice treated with Liq also had significantly fewer tumor nodules than control mice (Figure 5D). Immunohistochemical analysis of tumor sections revealed that Liq treatment resulted in less expression of the proliferation marker Ki-67 in tumors compared to control (Figure 5E). Further, the effect of Liq on apoptosis was confirmed by TUNEL assay. As shown in Figure 5F, the number of TUNEL-positive apoptotic cells was significantly greater in Liq treated tumors than in control tumors. To further study the effect of ERβ agonist Liq on the expression of NF-κB target genes, tumor sections were subjected to IHC analysis of IL-1 beta and Cox-2. As shown in Figure 5G, Liq treatment significantly reduced the expression of IL-1 beta and Cox-2 in tumors compared to vehicle treatment. Collectively, these results suggested that natural ERβ agonist significantly downregulated OCa progression *in vivo* and induced apoptosis.

## DISCUSSION

Emerging evidence suggests that ERβ functions as a tissue-specific tumor suppressor with anti-proliferative actions [23–26]. Despite ER expression in 67% of OCa, anti-estrogen therapy has had limited success, and the benefit of hormonal therapy has not been systematically studied [27]. ERβ expression is downregulated in many tumors including OCa [23, 26, 28–30]. Loss of ERβ expression increases the risk for metastasis [31], and correlates with shorter overall survival and the lack of clinical response to chemotherapy in OCa [32]. Reintroduction of ERβ in OCa cells reduces their proliferation [21]. Even though emerging evidence suggests a tumor suppressor role of ERβ in OCa, the clinical utility of ERβ is limited because of the lack of mechanistic insights and agents that specifically target ERβ.

We examined whether targeting ERβ using naturally available agonists has utility for treating OCa. Both S-equol and Liq had tumor suppressive functions by reducing OCa cell viability, migration and invasion. Further S-equol and Liq also inhibited the growth of therapy-resistant OCa cells and sensitized OCa cells to cisplatin and paclitaxel treatment. Our results support the data from earlier studies that reported ERβ agonists function as anti-cancer agents [26]. Recent study suggested that the high potency of Liq is due to the ability of the ERβ–Liq complex to recruit selective coactivators and its ability to bind to unique regulatory chromatin sites of estrogen-responsive genes [33]. Further, we provide evidence on the utility of Liq as a potential therapeutic agent using OCa Xenograft model.

Recent genomic studies suggested that ERα and ERβ have the potential to activate different sets of genes and that ERβ effects can be non-classical via its interactions with other transcription factors such as AP1, SP1, NF-κB and KLF5 [34, 35]. Global gene expression studies comparing estrogen and natural ERβ agonists revealed that natural ERβ agonists notably reduce the stimulation of genes promoting proliferation, and preferably induce genes that are more pro-apoptotic dependent on the status and ratio of ER subtypes present in a cell [36]. Our RNA-seq studies using OCa model cells confirmed these earlier observations and showed that ERβ natural agonists suppress cytokine, immune, and metastatic pathways and activate pathways that promote apoptosis.

Our RNA-seq analysis results found that majority of the top pathways modulated by Liq are related to inflammation. NF-κB pathway is the key mediator of many of the inflammatory processes. Recent studies suggest that NF-κB pathway plays essential roles in OCa initiation, progression, and its activation increases the aggressiveness of OCa [37, 38]. We found that treatment with Liq downregulated the expression of many genes involved in NF-κB pathway and negatively correlated with NF-κB signaling pathway and inflammation, further supporting the notion that Liq-mediated tumor suppression involves the attenuation of inflammatory pathways. NF-κB is known to regulate the expression of MMPs and is essential for the epithelial-mesenchymal transition and metastasis via modulation of MMPs [39, 40]. We found that Liq treatment suppresses the activation of MMPs. To corroborate these results, we examined the role of ERβ agonists in migration and invasion and found that both Liq and S-equol significantly reduced the migration and invasion of OCa cells.

ERβ exerts anti-inflammatory effects by repressing genes that promote inflammation, and ERβ signaling can cross-talk with NF-κB signaling pathway [7, 41–43]. Further, estrogens inhibit NF-κB binding to the IL-6 promoter, and ERβ-selective agonists inactivate microglia and invading T cells by downregulating the expression of NF-κB [44, 45]. The synthetic ERβ agonist DPN inhibited nuclear translocation and phosphorylation of p65 by inhibiting IκB degradation [46], and ERβ functions as a gate-keeper for NF-κB signaling by repressing its expression and nuclear translocation [47]. Accordingly, in mechanistic studies using NF-κB reporter assays, both S-equol and Liq significantly reduced the reporter activity. Further, we confirmed the down regulation of NF-κB target genes upon treatment with S-equol and Liq. More importantly, our study demonstrated that ERβ specifically interacts with p65 subunit of NF-κB and overexpression of ERβ reduced the expression of NF-κB target genes. Collectively, these findings suggest that natural ERβ agonists promote ERβ crosstalk with NF-κB and down regulate expression of NF-κB target genes.

In summary, the results from this study established the potential of two natural ERβ agonists for treating OCa and provided evidence that natural ERβ agonists promote tumor suppression and anti-inflammatory pathways by modulating gene expression. Since S-equol and Liq are currently in clinical trials for other clinical indications and are well tolerated, identification of ERβ agonist therapy as a novel therapeutic for OCa may be readily transferred to clinical use with current chemotherapies.

## MATERIALS AND METHODS

### Cell lines and reagents

Human OCa cell lines ES2, SKOV3, and BG-1 were obtained from the American Type Culture Collection (ATCC) and were maintained as per ATCC guidelines. All the cells were passaged in the user's laboratory for fewer than 6 months after receipt or resuscitation. For ERβ agonist's treatment, OCa cells were maintained in phenol red–free RPMI medium containing 5% dextran-charcoal treated FBS. S-equol was generously provided by Ausio Pharmaceuticals. Liquiritigenin (Liq) was purchased from Biopurify Phytochemicals (Chengdu, China). Paclitaxel was obtained from Tocris Bioscience (Bristol, UK). IL-1 beta, and p65 antibodies were purchased from Cell Signaling Technology (Beverly, MA), Cox-2 antibody was obtained from Santa Cruz Biotechnology (Dallas, TX) and FLAG antibody and Cisplatin were purchased from Sigma Chemical Co (St. Louis, MO). Turbofect transfection reagent and SuperScript III First Strand kit and luciferase lysis buffer were purchased from Thermo Scientific (Waltham, MA). *In situ* Cell Death Detection Kit was purchased from Roche (Indianapolis, IN). The Ki67 antibody was purchased from Abcam (Cambridge, MA). Stably ERβ-expressing ES2 cells were generated by infecting them with pLenti6/V5-D-FLAG ERβ or empty control vectors and positive cells were selected with blasticidine (5 μg/mL). The specificity of ERβ agonists, antibodies and expression vectors were validated in our previous studies [26, 48, 49].

### Cell viability, clonogenic and caspase-3/7 activity assays

The effect of ERβ agonists on OCa cell viability was assessed by using MTT assays. OCa cells were seeded in 96-well plates (2 × 10^3^ cells/well) and were treated with varying concentrations of S-equol or Liq for 72 h. The MTT reagent was added to each well and incubated for 4 h. Formazan crystals were solubilized in DMSO and the optical density was measured by using the micro-plate reader. The cell proliferation rates of control and ERβ-overexpressing ES2 cells were measured using MTT assay. For combination studies, ES2 cells were seeded in 96 well plates (1 × 10^3^ cells/well) and after an overnight incubation, the cells were pretreated with vehicle or Liq (25 μM) for 48 h followed by wash-off and replenished with varying concentrations of paclitaxel or cisplatin alone or in combination with Liq for 5 days. Cell viability was then measured using the MTT assay as described above. For the clonogenic assays, OCa cells (500 cells/well) were seeded in 6-well plates and after an overnight incubation, cells were treated with S-equol or Liq for 72 h. The cells were washed with PBS and replenished with culture media and allowed to grow for an additional 7 days. The cells were then fixed in ice-cold methanol and stained with 0.5% crystal violet solution to visualize the colonies. Colonies that contain ≥ 50 cells were counted. The effect of ERβ agonists on apoptosis was determined by using Caspase-3/7 activity assays. Briefly, 5 × 10^3^ cells were seeded in 96 well plates and after overnight incubation, cells were treated with S-equol (100 μM) or Liq (100 μM) for 48 h and the Caspase-3/7 activities were determined according to manufacturer protocol (Promega, Madison, WI).

### Cell migration and invasion assays

The cell migration and invasion were determined by using colorimetric QCM chemotaxis cell migration assay (EMD Millipore, Billerica MA) and the Corning^®^ BioCoat^™^ Growth Factor Reduced Matrigel Invasion Chamber assay, respectively. ES2 and SKOV3 cells were pretreated with S-equol (100 μM) or Liq (100 μM) for 24 h and subjected to cell migration and invasion assays according to manufacturer protocols.

### RNA sequencing and qRT-PCR

ES2 cells were treated with either vehicle or Liq (100 μM) for 24 h, and total RNA was isolated using RNAesy mini kit (Qiagen, Valencia, CA) according to the manufacturer's instructions. The purity of isolated RNA was determined by using Agilent 2100 BioAnalyzer. Illumina TruSeq RNA Sample preparation was performed following manufacturer's protocol, and the samples were run on an Illumina HiSeq 2000 (Illumina, Inc., San Diego, CA) in duplicates. RNA-sequencing was performed as described previously [50]. Differential expression analysis was performed by using DEseq, and significant genes with at least 1.5-fold change with p<0.05 were chosen for analysis. Using all significant and differentially expressed genes from the RNA-seq data, Ingenuity Pathway Analysis software (IPA) was used to interpret the biological pathways. To validate the selected genes, reverse transcription (RT) reactions were performed by using SuperScript III First-Strand kit, according to the manufacturer's protocol and real-time PCR was done using SYBR Green with the following primers: Actin: F-5′- GTGGGCATGGGTCAGAAG-3′; R-5′- TCCATCACGATGCCAGTG-3′ CXCL8: F-5′-ACTGAGAGTGATTGAGAGTGGAC-3′; R-5′-AACC CTCTGCCCCAGTTTTC-3′; MMP14: F-5′-CGAGGT GCCCTATGCCTAC-3′; R-5′-CTCGGCAGAGTCAAA GTGG-3′; MMP11: F-5′-CCGCAACCGACAGAAG AGG-3′; R-5′-ATCGCTCCATACCTTTAGGGC-3′; ICAM1: F-5′-ATGCCCAGACATCTGTGTCC-3′; R-5′-GGGGTCTCTATGCCCAACAA-3′; VCAM1: F-5′-CAG TAAGGCAGGCTGTAAAA GA-3′; R-5′-TGGAGCTG GTAGACCCTCG-3′; MMP1: F-5′-CTCTGGAGTAAT GTCACACCTCT-3′; R-5′-TGTTGGTCCACCTTTCATC TTC-3′; PIK3CG: F-5′-GGCTACCATGAGCAGCTTA CC-3′; R-5′-CTGTGAGGTCGGTGTTCCG-3′; TNF: F-5′-CCTCTCTCTAATCAGCCCTCTG-3′; R-5′-GAGGA CCTGGGAGTAGATGAG-3′; MMP3: F-5′-TTCACTC AGCCAACACTG AA-3′; R-5′-ACAGCATCAAAGG ACAAAGC-3′; IL-1 beta: F-5′-AAGCTGAGGAAG ATGCTG-3′; R-5′-ATCTACACTCTCCAGCTG-3′; PTGS2: F-5′-CTGGCGCTCAGCCATACAG-3′; R-5′-CGCACTTATACTGGTCAAATCCC-3′. Results were normalized to β-actin transcript levels, and the difference in fold expression was calculated using delta-delta-CT method.

### Reporter gene assays

ES2, SKOV3, and SKOV3-TR cells were maintained in phenol red–free RPMI supplemented with 5% dextran coated charcoal–striped serum for 48 h prior to transfection. Cells were transiently transfected with 250 ng of NF-κB-Luc reporter plasmid using Turbofect transfection reagent. After 24 h, cells were treated with vehicle, S-equol, or Liq for additional 24 h. Renilla reporter (50 ng) plasmid was co-transfected and used for data normalization. Cells were lysed in Luciferase Lysis Buffer, and the luciferase activity was measured by using the luciferase assay system (Promega, Madison, WI) in a luminometer.

### Immunoprecipitation and western blotting

ES2 cell lysates were prepared using NP40/TritonX100-lysis buffer (50 mM Tris·HCl at pH 7.5, 0.2% Triton X-100, 0.3% Nonidet P-40, 150 mM NaCl, 25 mM NaF, 0.1 mM sodium orthovanadate) containing protease and phosphatase inhibitors. Cell lysates were immunoprecipitated with FLAG antibody, followed by Western blotting with p65 antibody. For GST pulldown assays, lysates were incubated with GST or ERβ-GST fusion proteins, and bound proteins were isolated by GST-pull-down assay, and the interaction of ERβ with p65 was analyzed by Western blotting. Total proteins or IP complexes were mixed with 4X SDS sample buffer and subjected to SDS-PAGE. Resolved proteins were then transferred onto nitrocellulose membranes and subjected to blocking with 5% non-fat dry milk powder for 1 h at room temperature and incubated with primary antibodies over night at 4°C followed by incubation with secondary antibody for 1 h at room temperature. Blots were developed using the ECL kit (Thermo Scientific, Waltham, MA).

### Nude mice studies

All animal experiments were performed after obtaining UTHSCSA IACUC approval, and all the methods were carried out in accordance with the IACUC-approved guidelines. SKOV3ip1 cells labelled with GFP-Luciferase (1 × 10^6^ cells) were injected intra-peritoneally into 6-8 weeks old female athymic nude mice as described previously [51]. Tumor progression in mice was monitored by using Xenogen *in vivo* imaging system. Mice were randomly assigned into two groups (n = 5 mice per group): (a) control (30% Captisol) (b) Liq (20 mg/Kg body weight/day). The mice were monitored daily for adverse effects, and the treatment was continued for 3 weeks. At the end of the treatment, tumor weight, and the number of nodules were recorded. The tissue samples were fixed in formalin and processed for histological studies.

### Immunohistochemistry

Immunohistochemical analysis was performed as described previously [26]. Tumor tissue sections were incubated in xylene and passed through series of graded alcohols followed by antigen retrieval using the antigen retrieval solution (Vector Labs, Burlingame, CA). Tissue sections were incubated in 3% H_2_O_2_ solution for 20 min and then subjected to blocking using the Vector Lab Blocking Kit. Tissue sections were incubated overnight with primary antibodies of Ki-67 (1:100), Cox-2 (1:50), and IL-1 beta (1:100) and then with secondary antibodies. Immunoreactivity was visualized by using the DAB substrate and counterstained with hematoxylin (Vector Lab, Inc.). The proliferative index was calculated as percentage of Ki-67-positive cells in five randomly selected microscopic fields. TUNEL analysis was performed using the *In situ* Cell Death Detection Kit according to manufacturer's protocol and DAPI was used to visualize the nuclei. Percentage of apoptotic cells were calculated from five randomly selected microscopic fields in each group.

### Statistical analysis

GraphPad Prism 6 software (GraphPad Software, SanDiego, CA) was used for all statistical analyses. A Student's t-test was used to assess statistical differences between control and Liq-treated groups. All the data represented in bar graphs are shown as means ± SE. Statistical differences among groups were analyzed with ANOVA. *P* value less than 0.05 was considered significant.
